# Salvage Hypofractionated Radiotherapy in a Progressive Radiation-Induced Angiosarcoma: A Case Report

**DOI:** 10.7759/cureus.1886

**Published:** 2017-11-28

**Authors:** Elisabetta Bonzano, Marina Guenzi, Renzo Corvò

**Affiliations:** 1 Radiation Oncology, University of Genoa Ospedale Policlinico San Martino; 2 Radiation Oncology, University of Genoa Ospedale Policlinico San Martino

**Keywords:** breast, radiation-induced, angiosarcoma, nodal progression, radiotherapy, case report

## Abstract

We report a case of a long-term local control and survival achieved in a patient affected by radiation-induced angiosarcoma (RIA).

A 57-year-old woman had a history of breast-conserving surgery and radiation therapy for primary breast cancer. Eight years after the mastectomy, multiple nodal progression was diagnosed as RIA and subsequentially treated by salvage lymph node dissection followed by adjuvant intensified radiotherapy to control the residual disease. Two and a half years later, the patient is alive and cancer-free.

This experience shows that radiotherapy may have the potential to be a feasible and effective treatment to control RIA progression, and it may also play a role in the management of RIA as adjuvant.

## Introduction

Angiosarcoma of the breast is a rare endothelial malignant tumor; it may appear spontaneously, may be radiation-induced, or occur following axillary lymph node dissection (ALND) for mammary carcinoma as a consequence of chronic lymphedema [1]. A careful analysis of already published case reports on this topic can be essential for choosing the most effective treatment [2]. We briefly report the management of an aggressive angiosarcoma developing in a breast that was treated with surgery, plus radiotherapy, for a ductal carcinoma in situ (DCIS) eight years before. Because of the latency intercurring, it could be reasonably stated as radiation-induced.

## Case presentation

**Figure 1:**
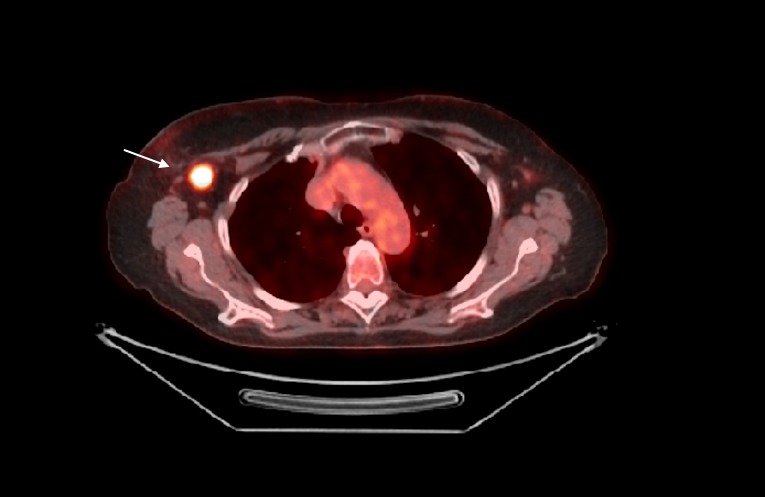
PET/CT scan shows a contralateral recurrence of the angiosarcoma (white arrow) PET/CT: positron emission tomography/computed tomography

In March 2006, a 57-year-old woman affected by a DCIS (solid and cribriform type, pTisN0) underwent a left breast lumpectomy and sentinel lymph node dissection, followed by radiotherapy. The patient received conventional whole breast irradiation, 50 Gy in 25 daily fractions delivered over five weeks (200 cGy/ fraction). According to the histological characteristics, no systemic therapy was prescribed. After a disease-free interval of eight years, in June 2014, the patient presented with a palpable, painless, increased consistency, red skin lump in the central portion of the left breast. One month later, a diagnostic biopsy showed an immune-phenotypic histology suggestive of angiosarcoma. Therefore, in August 2014, the patient underwent a left breast mastectomy. Histopathological examination revealed a moderately differentiated angiosarcoma infiltrating the cutaneous left breast tissue with a mass of 10 cm in its largest diameter and localized in the superior and central portion of the left breast. In the absence of a widely accepted consensus, no local adjuvant treatment was performed. The patient started a clinical and diagnostic three-month follow-up. In January 2015, the ultrasonography examination revealed two enlarged lymph nodes in the right axilla. Chest and abdominal computed tomography (CT) confirmed an early contralateral recurrence of the angiosarcoma, also detected by a fluorine-18 fluorodeoxyglucose (FDG) positron emission tomography/computed tomography (PET-CT) (Figure 1). 

**Figure 2:**
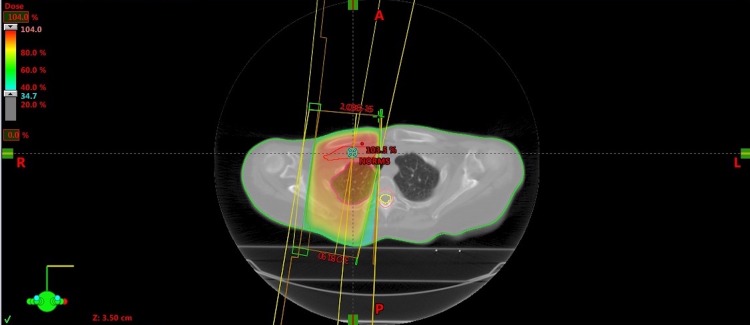
Dose plan

In February 2015, the patient underwent a right ALND that revealed that 2/15 lymph nodes were involved with massive metastasis. Therefore, in March 2015, the patient received adjuvant chemotherapy consisting of an intravenous infusion of paclitaxel, 155 mg one hour weekly, for six cycles. Twenty-one days later, the patient started adjuvant right third axillary level and supraclavicular regional radiotherapy. Written informed consent was obtained before treatment. According to our institutional protocol, the radiation treatment schedule consisted of a mild hypofractionation: 46 Gy delivered in 2.3 Gy daily fractions four times a week in 20 fractions, which is biologically comparable to 50 Gy in 25 fractions for five weeks. Radiotherapy was precisely delivered to the high-risk right area of her previous dissection (Figure 2).

**Figure 3:**
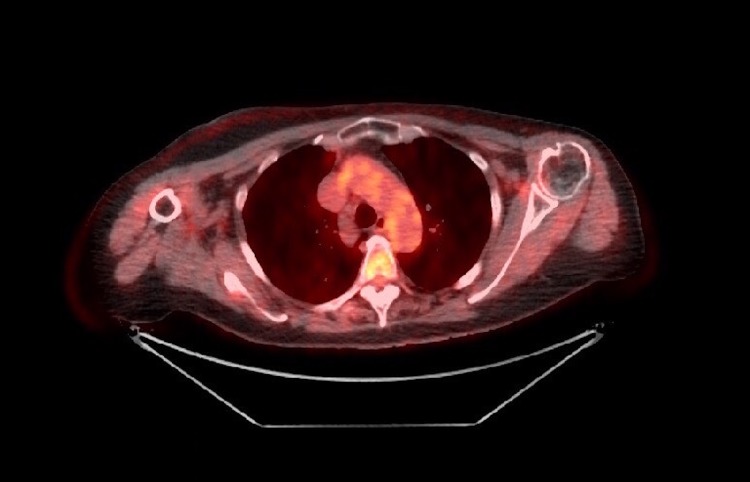
PET/CT negative for nodal disease after multimodality treatment PET/CT: positron emission tomography/computed tomography

No fibrosis sequel was seen and only mild erythema occurred after the radiation treatment. Subsequently, the patient has been followed with routine clinical and imaging examinations at four-month intervals. Currently, a 30-month follow-up examination revealed a complete remission (Figure 3).

## Discussion

As radiation-induced angiosarcoma (RIA) occurrence is rare (cumulative incidence about 0.9 per 1,000 cases during 15 years) [3], we cannot consider the risk of cancerogenesis being able to exceed the benefit of adjuvant radiotherapy for breast cancer [4]. Typically, RIA occurs in older women and is characterized by the following three features: location in the previous field of radiation, a latency of years after radiation therapy, and lastly, a histologic distinction from the primary neoplasm [5]. As described in our case, and in accordance with the current literature, immediate surgery followed by adjuvant histology-driven treatments (taxane derivatives) represents the therapeutic stronghold against angiosarcomas [6]. Our experience also sheds light on the essential role of the histological type. Our patient was affected by a moderately differentiated angiosarcoma, expressing positive immunohistochemistry for CD31, CD34, Factor VIII, and a high Ki-67 growth fraction index. Expression of CD31 is considered the most useful marker in the diagnosis of poorly differentiated angiosarcomas due to its high sensitivity and specificity [7]. Furthermore, CD34 and Factor VIII are expressed in most angiosarcomas but less in poorly differentiated tumors [8]. Currently, taxanes and anthracyclines are considered the most effective therapies for aggressive angiosarcomas [9]; however, novel targeted therapies are being studied [10]. Despite the intrinsic radioresistance of angiosarcomas, radiotherapy is widely suggested both with curative and palliative intents in advanced disease [1].

## Conclusions

In our case, we chose a hypofractionated radiation treatment with the aim to counteract radioresistance. This treatment, targeted at the high-risk area of the microscopic angiosarcoma, has proven to be well-tolerated and effective. At our patient's 30-month follow-up, neither radio-induced toxicity nor evidence of short-term relapse was observed. In conclusion, our experience shows that radiotherapy could be a valid and appropriate treatment to control RIA progression and may also have a role in the management of RIA also as an adjuvant.
